# Intramolecular Hydrogen Bonding 2021

**DOI:** 10.3390/molecules26206319

**Published:** 2021-10-19

**Authors:** Mirosław Jabłoński

**Affiliations:** Faculty of Chemistry, Nicolaus Copernicus University in Toruń, Gagarina 7, 87-100 Toruń, Poland; teojab@chem.umk.pl; Tel.: +48-056-611-4695

Undoubtedly, hydrogen bonds occupy a leading place in the rich world of intermolecular interactions. This fact results from their intermediate strength, being between stronger covalent bonds and weaker van der Waals interactions. For this reason, intermolecular hydrogen bonds act as a glue that binds individual molecules into dimers or larger molecular clusters, but at the same time allows for dynamic changes consisting in breaking old bonds and possible formation of new ones. This ability is partly due to the small size of the hydrogen atom, which, being poor in electron density, allows the X and Y units in the X-H…Y contact to come closer to each other with relatively small steric effects. The most frequently quoted academic symptoms of the presence of intermolecular hydrogen bonding are the unexpectedly high boiling point of water, lower density of ice compared to liquid water, and the good solubility of polar and ionic substances in water. Nevertheless, equally important is the presence of hydrogen bonds between the complementary nitrogen bases (C-G, A-T) in the nucleotides of two DNA strands, because it leads to a double-helical structure that is fundamental in the replication of genetic information. Intermolecular hydrogen bonds also have a huge impact on the formation of the structure of proteins or the maintenance of cellulose and polymer chains.

It seems that intramolecular hydrogen bonds are described somewhat less frequently. The reason may be the problem of direct detection of intramolecular hydrogen bonds, which in turn entails the need to use some indirect methods, both experimental and theoretical. This generally requires finding a reasonable reference system, which is often not so trivial. Like their intermolecular counterparts, intramolecular hydrogen bonds are also often of great importance. They significantly influence the stability and physico-chemical properties of the conformers in which they occur.

The year 2021 will be known historically as the year of the SARS-CoV-2 coronavirus, which has made scientific research difficult for many. However, despite these difficulties, this Special Issue has collected as many as 12 articles, including 4 reviews. Thus, this has to be seen as a success, all the more so as these articles deal with many issues of intramolecular hydrogen bonding. Moreover, these articles gather both experimental and theoretical results. At the same time, the rich subject matter of the articles of this Special Issue proves that intramolecular hydrogen bonds are, despite over a hundred years of investigations in this field, still an important object of scientific research.

Two of the four review articles are devoted to theoretical methods for estimating the energy of intramolecular interactions, including, of course, hydrogen bonds [1,2]. In the first of them, written by Jabłoński [1], the main goal was to present the theoretical rationale of a given method and to show that often many variants of a given method are possible. This generally leads to a significant range of the estimate values obtained. The limitations of the methods used are also discussed. This review is then brilliantly complemented by Deshmukh and Gadre in their review on the Molecular Tailoring Approach [2]. Importantly, this fragmentation method allows the energy of an individual intramolecular hydrogen bond to be estimated in systems containing many such interactions. The authors present many examples of the use of this method.

Hansen [3] reviews intramolecular NH…X (X = O, S, N) bonds in various systems and describes the usefulness of spectroscopic parameters (mainly based on NMR) in assessing their strength. He points out that the NH chemical shift should be corrected for ring current effects when the substituent at the nitrogen is an aromatic ring. The importance of the two-bond deuterium isotope effects (TBDIE) on ${}^{13}$C chemical shifts is then underlined.

Studies on excited states are usually regarded as more problematic than ground-state studies and are therefore much rarer. For this reason, the review by Jankowska and Sobolewski on the excited-state intramolecular proton transfer (ESIPT) is particularly important [4]. These authors discuss the modern theoretical methods of the ESIPT description, paying attention to their range of applications and limitations.

The remaining articles are good evidence, showing that in order to study the phenomenon of hydrogen bonding, many different research techniques, both experimental and theoretical, are used. For example, Filarowski and collaborators [5] use infrared (IR) and Raman spectroscopy, incoherent inelastic neutron scattering (IINS), X-ray diffraction, nuclear quadrupole resonance spectroscopy (NQR), differential scanning calorimetry (DSC) along with DFT calculations to explain the influence of the O-H…O intramolecular hydrogen bond on the polymorphic states and isomerization of 5-chloro-3-nitro-2-hydroxyacetophenone. In a similar way, Tolstoy, Filarowski and collaborators [6] also investigate the intramolecular O-H…N hydrogen bond in 2-[(E)-(phenylimino)methyl]phenol, which is one of the most photo-thermochromic compounds. Their spectroscopic studies confirm that this bond can be classified as a resonance-assisted hydrogen bond (RAHB).

Alkhimova, Babashkina and Safin [7] investigate solvatochromic and luminescent properties of four *N*-salicylidene aniline derivatives derived from the ethyl ester of glycine, whose photophysical properties are dictated by the intramolecular proton transfer in the O-H…N bridge. It is shown that the physicochemical properties of these compounds, related to a subtle keto-enamine-enol-imine equilibria, can be tuned by the nature of the solvent used (non-polar vs. polar aprotic vs. polar protic).

Many methods (including Car-Parrinello Molecular Dynamics (CPMD) for the gas and crystalline phases) are used by Jezierska, Panek and collaborators in their theoretical studies of 2,3-dimethylnaphthazarin and 2,3-dimethoxy-6-methylnaphthazarin [8]. These authors show that the proton transfer phenomenon takes place in both the compounds as well as in both phases. Importantly, the nuclear quantum effects (NQE) are shown to localize the proton closer to the half of the O…O contact. Nevertheless, NQE should have no qualitative impact on the properties of the investigated molecules. The strong mobility of the bridged protons is also confirmed by spectroscopic data.

Alkorta, Elguero and Del Bene [9] investigate intramolecular O-H…O hydrogen bond in 1-oxo-3-hydroxy-2-propene (i.e., 3-hydroxy-2-propenal or simply the enol form of malondialdehyde) and the change of its characteristics during interaction of this molecule with Lewis acids LiH, LiF, BeH${}_{2}$, and BeF${}_{2}$. They found that the binding of these acids to the -C=O group is more preferred than to -OH and that ${}^{2h}$J(O-O), ${}^{1h}$J(O-H), and ${}^{1h}$J(H-O) spin–spin coupling constants exhibit a second-order dependence on the O…O, O-H, and H…O distances, respectively.

Lamsabhi, Mó, and Yáñez [10] utilize the high-level ab initio G4 theory to study the O-H…N intramolecular hydrogen bond in a series of the most stable conformers of HOCHX(CH${}_{2}$)${}_{n}$CH${}_{2}$NH${}_{2}$ and HOCH${}_{2}$(CH${}_{2}$)${}_{n}$CHXNH${}_{2}$ (*n* = 0–5) where X is H, F, Cl, or Br substituted in position α with respect to either -OH or -NH${}_{2}$. The strongest hydrogen bond occurs when *n* = 2 as shown by shortest H…N distance, isodesmic reaction-based largest interaction energy, largest red-shift of $\nu_{\mathrm{OH}}$, and NBO, QTAIM, and NCI theoretical methods. In the group of substituents X, Br gives the greatest influence on OH…N, but interestingly, it is the opposite depending on whether this substituent is in position α with respect to -OH or with respect to -NH${}_{2}$. This article [10] also investigates the effect of interaction with the BeF${}_{2}$ molecule.

Intramolecular O-H…O hydrogen bond in malonaldehyde is also theoretically investigated by Pendás and collaborators [11]. Additionally, the influence of eight substituents (both electron-withdrawing and electron-donating) at each of the three skeletal carbon atoms is investigated, and then the OH…O energy is determined using the proprietary IQA method and compared with their equivalents obtained using the OCM and EM methods (see also [1]). While in general the O-H…O bond can either be weakened or strengthened depending on the substituent and the site of substitution, the substitution next to -OH always significantly strengthens this bond (see also [10]). It turns out that for the tested RAHB systems, IQA energies correlate well with EM energies, while there is no such correlation with OCM.

Noticeably, using Local Mode Analysis (and QTAIM and NCI), Altun, Bleda and Trindle [12] order the various intramolecular hydrogen bonds present in tautomers and isomers of 3-hydroxy-2-butenamide according to their strength as follows: the strongest O-H…O=C > N-H…O=C > O-H…N, intermediate N-H…O=C ≥ N-H…O ≈ C-H…O=C, the weakest C-H…N > C-H…O.

## References

[1] Jabłoński M. (2020). A Critical Overview of Current Theoretical Methods of Estimating the Energy of Intramolecular Interactions. Molecules.

[2] Deshmukh M.M., Gadre S.R. (2021). Molecular Tailoring Approach for the Estimation of Intramolecular Hydrogen Bond Energy. Molecules.

[3] Hansen P.E. (2021). A Spectroscopic Overview of Intramolecular Hydrogen Bonds of NH…O, S, N Type. Molecules.

[4] Jankowska J., Sobolewski A.L. (2021). Modern Theoretical Approaches to Modeling the Excited-State Intramolecular Proton Transfer: An Overview. Molecules.

[5] Hetmańczyk Ł., Szklarz P., Kwocz A., Wierzejewska M., Pagacz-Kostrzewa M., Melnikov M.Y., Tolstoy P.M., Filarowski A. (2021). Polymorphism and Conformational Equilibrium of Nitro-Acetophenone in Solid State and under Matrix Conditions. Molecules.

[6] Hetmańczyk Ł., Goremychkin E.A., Waliszewski J., Vener M.V., Lipkowski P., Tolstoy P.M., Filarowski A. (2021). Spectroscopic Identification of Hydrogen Bond Vibrations and Quasi-Isostructural Polymorphism in N-Salicylideneaniline. Molecules.

[7] Alkhimova L.E., Babashkina M.G., Safin D.A. (2021). A Family of Ethyl *N*-Salicylideneglycinate Dyes Stabilized by Intramolecular Hydrogen Bonding: Photophysical Properties and Computational Study. Molecules.

[8] Kułacz K., Pocheć M., Jezierska A., Panek J.J. (2021). Naphthazarin Derivatives in the Light of Intra- and Intermolecular Forces. Molecules.

[9] Alkorta I., Elguero J., Del Bene J.E. (2021). Perturbing the O–H⋯O Hydrogen Bond in 1-oxo-3-hydroxy-2-propene. Molecules.

[10] Lamsabhi A.M., Mó O., Yáñez M. (2021). Perturbating Intramolecular Hydrogen Bonds through Substituent Effects or Non-Covalent Interactions. Molecules.

[11] Guevara-Vela J.M., Gallegos M., Valentín-Rodríguez M.A., Costales A., Rocha-Rinza T., Pendás A.M. (2021). On the Relationship between Hydrogen Bond Strength and the Formation Energy in Resonance-Assisted Hydrogen Bonds. Molecules.

[12] Altun Z., Bleda E.A., Trindle C. (2021). Focal Point Evaluation of Energies for Tautomers and Isomers for 3-hydroxy-2-butenamide: Evaluation of Competing Internal Hydrogen Bonds of Types -OH…O=, -OH… N, -NH…O=, and CH…X (X=O and N). Molecules.

